# Interventions to Optimise Mental Health Outcomes During the COVID-19 Pandemic: A Scoping Review

**DOI:** 10.1007/s11469-021-00558-3

**Published:** 2021-06-15

**Authors:** Jacqueline Safieh, John Broughan, Geoff McCombe, Niamh McCarthy, Timothy Frawley, Allys Guerandel, John S. Lambert, Walter Cullen

**Affiliations:** 1 School of Medicine, University College Dublin, Dublin, Ireland; 2 Seanoira Day Hospital, St. Camillus Hospital, Limerick, Ireland; 3 Department of Psychiatry and Mental Health Research, St. Vincent’s University Hospital, Dublin, Ireland; 4 School of Nursing, Midwifery, and Health Systems, University College Dublin, Dublin, Ireland; 5 Ireland East Hospital Group, Dublin, Ireland; 6 Department of Infectious Diseases, Mater Misericordiae University Hospital, Dublin, Ireland

**Keywords:** COVID-19, Pandemic, Mental health, Well-being, Interventions

## Abstract

Adverse mental health has been a major consequence of the COVID-19 pandemic. This review examines interventions to enhance mental health outcomes and well-being of populations during COVID-19. Four electronic databases (MEDLINE, PsycINFO, Embase, and CINAHL) were searched following Arskey and O’Malley’s six-staged scoping review process. Twenty studies were included in the review. Various study populations were included to ensure greater generalisability of results. Interventions informing treatment of mental health concerns during COVID-19 were included and classified into (a) prevention of poor mental health, (b) therapeutic interventions, and (c) other interventions. Preventative strategies (n = 16) included public health education, modified social media use, technology-based interventions, physical activity, policy adaptations, and therapeutic interventions. Treatment strategies (n = 7) included adapting existing treatment and the creation new treatment programmes and platforms. While current evidence is promising, future research should focus on novel effective interventions to address mental health issues during the pandemic.

## Background

In January 2020, the World Health Organization (WHO) declared the outbreak of novel coronavirus SARS-CoV-2, responsible for COVID-19, to be a public health emergency of international concern. Public health experts and officials worked quickly at the WHO and around the world to contain the disease and protect populations. However, as with past epidemics and pandemics, the emergence of the pandemic brought psychological and mental health responses including the spread of fear, stress, and anxiety, which also impact the spread and containment of infectious diseases (Taylor 2019). Early research into mental health during COVID-19 thus far has demonstrated that there may be a considerable degree of psychological and mental health impacts from COVID-19, affecting whole populations. It is therefore crucial to address the public health crisis of mental health during the COVID-19 pandemic.

Pandemics such as COVID-19 often result in large disruption to populations, which may account for the increased population mental health burden (Cullen et al. 2020). Some of the disruptions that influence mental health include uncertainty (regarding who is at risk or if the pandemic is truly over), confusion, and a sense of urgency (Taylor 2019). A relatively newer phenomenon that has been observed during the COVID-19 pandemic is the role of social media in providing information, but also misinformation, which may be linked to changes in mental health (Gao et al. 2020; Taylor 2019). Other factors that may affect mental health include health threats (to self and loved ones); disruptions to routines; quarantine and social distancing (Brooks et al. 2020); financial setbacks due to loss of work, and shortages of food, medicines, and other essential items; exposure to death of self and loved ones; inability to practice common religious and cultural rituals around mourning the deceased; and indirect exposure to trauma (Taylor 2019). Additionally, while this paper will focus on the mental health impacts on the general population, it is also worth noting that certain groups may have unique stressors, for example cultural minorities, new immigrants, and refugees may be unfamiliar with community supports, have difficulties accessing services due to language barriers, and face discrimination (Júnior et al. 2020; Taylor 2019). A review of psychological responses during past pandemics found that often pandemics have been associated with an increase in ‘anxiety/fears, depression, anger, guilt, grief and loss, post-traumatic stress, and stigmatisation’ (Chew et al. 2020).

Studies have demonstrated significant mental health burdens in regions affected by COVID-19 outbreaks, in Asia (Ahorsu et al. 2020; Huang and Zhao 2020; Lei et al. 2020; Liu et al. 2020d), Europe (Cellini et al. 2020; González-Sanguino et al. 2020; Ozamiz-Etxebarria et al. 2020; Reznik et al. 2020; Soraci et al. 2020), North America (Lee 2020; Taylor et al. 2020), and Africa (Arafa et al. 2021). Some studies have also identified and analysed groups at a higher risk for developing poor mental health during COVID-19, including healthcare workers (Bansal et al. 2020; Lu et al. 2020; Rossi et al. 2020; Zhu et al. 2020), elderly (Hayek et al. 2020; Lopez et al. 2020), adolescents and youth (Liang et al. 2020; Zhou et al. 2020), and individuals with pre-existing mental illnesses (Li and Zhang 2020; Liu et al. 2020a; Moore et al. 2020). Other high-risk groups that have been identified by experts, but have not yet been rigorously studied include homeless individuals, individuals of lower socioeconomic status, racialised individuals, females, and refugees (Fortuna et al. 2020; Júnior et al. 2020; Lund 2020; Swinford et al. 2020; Tsai and Wilson 2020; Wenham et al. 2020).

Given the decrease in mental health amongst populations as a result of COVID-19 and associated safety measures (such as physical distancing and quarantine (Brooks et al. 2020), this scoping review aims to identify potential interventions that may be useful at addressing the rise in mental health presentations in the general population, especially given the unique circumstances around COVID-19.

## Methods

Scoping review methodologies are a popular adopted approach particularly in research fields which are rapidly evolving with emerging evidence (Anderson et al. 2008; Arksey and O’Malley 2005), as is the case for COVID-19. Scoping reviews are particularly valuable as a form of research synthesis with the goal to ‘map the literature on a particular topic or research area and provide an opportunity to identify key concepts; gaps in the research; and types and sources of evidence to inform practice, policymaking, and research’ (Daudt et al. 2013). In this context, a scoping review was the preferred methodology, as it provides an overview and synthesis of a broad or rapidly emerging topic, as is the case with research relating to COVID-19.

The scoping review methodological framework, comprising of an iterative six stage process (Arksey and O’Malley 2005), was undertaken in this review. This approach was taken to provide a comprehensive and systematic search of the current literature regarding interventions for improving or preventing decline in population mental health during the global COVID-19 pandemic. The six stages of the scoping review process are as described below.

### Identifying the Research Questions

This review aimed to identify current interventions for improving or preventing decline in population mental health during the global COVID-19 pandemic. The research question was, ‘what interventions have been used to improve population mental health during the COVID-19 pandemic?’

Interventions were considered to be ‘any activity undertaken with the objective of improving human health by preventing disease, by curing or reducing the severity or duration of an existing disease, or by restoring function lost through disease or injury’, including public health and clinical care measures (Smith et al. 2015). A wide definition of ‘interventions’ was used, as the literature in this field is still emerging, and the goal of the study was to map any methods that have been employed thus far.

The definition of negative mental health outcomes used was also quite broad, encompassing any deviation from ‘a state of well-being that allows individuals to cope with the normal stresses of life and function productively’ (Fusar-Poli et al. 2020). Primary mental health outcomes that were assessed and included in this review included symptoms of fear, stress, poor sleep, anxiety, and depression. Papers documenting individuals with pre-existing and recently developed symptoms of mental health issues were also included in the study.

### Identifying Relevant Studies

**Fig. 1:**
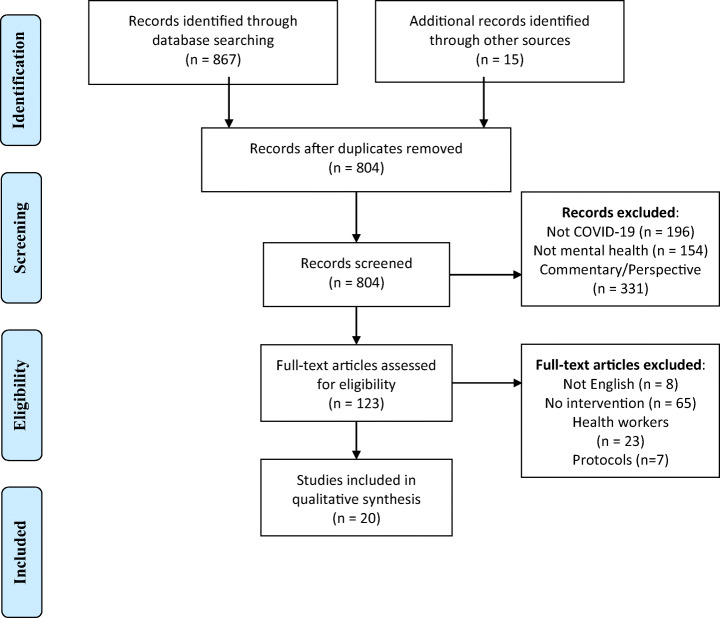
PRISMA flow chart of included studies

From June 1 to June 10, 2020, four electronic databases (MEDLINE, PsycINFO, Embase, and CINAHL) were searched. The search was briefly updated July 1– 5, 2020, to account for the rapidly evolving nature of COVID-19 research. Searches were conducted using a pre-determined search strategy. This search strategy was composed of an arrangement of terms linking concepts of mental health and COVID-19 (see Table 1). Search results were incorporated into a data management programme (EndNote). Articles were removed in three stages, following PRISMA guidelines (Moher et al. 2009); first duplicates were removed using EndNote software, next title and abstracts were screened, and finally full texts were screened (see Fig. 1). Two reviewers (JS + JB) screened the articles, and disagreements were discussed. In addition, databases of COVID-19 literature were hand searched. These databases included CORD-19, MedArchives, Zenodo, and WHO COVID-19 Research Database. The references of articles that matched the eligibility criteria were also further searched. The studies obtained through reference searching and through the COVID-19 literature databases were subject to the same screening and selection process.

**Table 1:** MEDLINE Search Strategy combining pandemic and mental health outcomes

[Ovid MEDLINE(R) and Epub Ahead of Print, In-Process & Other Non-Indexed Citations, Ovid MEDLINE(R) Daily 1946 to Present]	
1. Mental Health/ 2. Depression 3. Anxiety 4. Trauma* 5. Paranoia* 6. OCD 7. Obsessive compulsive disorder* 8. Phobia* 9. Wellbeing 10. Mental health 11. Psych* 12. 1 or 2 or 3 or 4 or 5 or 6 or 7 or 8 or 9 or 10 or 11	
13. Coronavirus/ 14. Coronavirus Infections/ 15. Pandemics/ 16. Disease Outbreaks/ 17. Pandemic 18. Covid* 19. Coronavirus* 20. 13 or 14 or 15 or 16 or 17 or 18 or 19	
21. 12 and 20

### Study Selection

The studies selected for inclusion were identified primarily through the search strategy outlined for the various databases, with few additional studies identified from hand-searching key literature (see Fig. 1). No restrictions were placed on the types of interventions included, nor on the timing of implementation of interventions (i.e. some interventions were implemented at the start of the pandemic, while others were implemented later on). Interventions were included that informed prevention or treatment of mental health concerns during COVID-19, including the following: (a) prevention of symptoms of poor mental health, (b) therapeutic interventions, (c) other interventions including legislation and health systems. Studies of all design types were eligible for inclusion if they were published in English from 2019 onwards, presented primary data, and the primary focus was on mental health during the COVID-19 pandemic. Broad inclusion criteria without assessment of methodological quality facilitated the inclusion of a wider pool of literature, including quantitative, qualitative, and mixed-method studies, as well as review articles. Protocols were excluded. Since the study focused on mental health of the general population, the only limitation placed on the study population was to exclude healthcare workers, as this was considered a unique population that may be investigated separately in subsequent studies.

### Charting the Data

Once all exclusion criteria were applied the data from the remaining studies were charted. The data from included articles were organised into a table to facilitate comparison and thematic analysis (Table 2), using the following headings: author, year; location; study design; population; intervention, control; and principle outcome measures.

**Table 2:** Characteristics of included studies

Author, year	Location	Study design	Population	Timing of intervention	Setting	Preventative/treatment	Intervention, control	Principle outcome measures
Ahmad and Murad 2020 [45]	Iraqi Kurdistan	Cross-sectional (online survey)	Public–social media users ≥18years (n = 516)	Start of pandemic	Community	Prevention (behaviour change, education)	Social media exposure	• Panic • Anxiety
Banskota et al. 2020 [51]	Global (US based)	Narrative review	Older adults	Not specified	Community	Prevention (behaviour change, education) and treatment (telemedicine)	Smartphone apps [(1) social networking; (2) medical, with subcategories (a) telemedicine and( b) prescription management; (3) health and fitness; (4) food and drink; and (5) visual and hearing impairment.]	• Functioning of apps • Ranking
Brooks et al. 2020 [57]	Global (UK based)	Rapid review	Public	All stages (during and post quarantine)	Community	Prevention	Effective rapid communication, supplies, shorter quarantine, appeals to altruism	• Psychological effects of quarantine
Cheng et al. 2020 [63]	Wuhan, China	Qualitative	COVID-19 inpatients (n = N/A)	Not specified	Hospital	Treatment	Web-based psychological first aid -rapid assessment and treatment sorting -online support and education groups for patients and staff	• Self-reported experiences with the intervention
Gao et al. 2020 [3]	China	Cross-sectional (online survey)	Public–aged ≥18years (n = 4872)	Jan 31–Feb 2, 2020 (approaching peak)	Community	Prevention (behaviour change, education)	Social media exposure	• Social media exposure (SME) • Depression: WHO-Five Well-Being Index (WHO-5) • Anxiety: generalised anxiety disorder scale (GAD-7)
Goethals et al. 2020 [54]	France	Qualitative	Older adults ≥60years (n = 6) and professionals running programme (n = 8)	Not specified (likely at peak)	Community	Prevention (behaviour change)	Physical activity (participation in French Federation of Physical Education and Voluntary Gymnastics (FFPEVG)	• Physical and mental health
Goodman-Casanova et al. 2020 [46]	Spain	Cross-sectional (telephone)	Older adults with mild dementia (n = 93)	March 25–April 6, 2020 (peak)	Community and specialist care	Prevention and treatment	Television-based assistive integrated technology, TV-AssistDem	• Health status • Mental health and well-being • Sleep quality
Huang et al. 2020 [60]	Zhejiang, China	Case report	COVID-19 patient; pregnancy and early postpartum (n = 1)	Feb 7, 2020–Feb. 19, 2020	Hospital	Treatment	Dialectical behavioural therapy (DBT)	• Chinese versions of Hamilton Depression Scale-18 (HAMD-17) • Motgomery-Asberg Depression Scale (MADRS) • Hamilton Anxiety Scale (HAMA)
Jacobson et al. 2020 [58]	USA	Longitudinal (during beginning of outbreak)	Public	March 16–23, 2020 (start)	Policy	Prevention	Stay-at-home orders	• Mental health search terms on Google
Kwon et al. 2020 [64]	Korea	Case report	COVID-19 patients in community settings -individuals self-contained in contact with confirmed patient	Not specified	Community	Treatment	Telemedicine and mind-body modalities (mindfulness meditation) - mental health instruction manual in telemedicine for doctors	• Anxiety, depression, fear and anger • Physical symptoms: pain, digestive problems, insomnia
Liu et al. 2020a [59]	Hainan, China	Randomised controlled trial	COVID-19 patients in isolation ward (n = 51)	January 1–February 16, 2020	Hospital (ICU)	Prevention	Progressive muscle relaxation (PMR) (30 min/day for 5 consecutive days)	• Spielberger State-Trait Anxiety Scale (STAI) • Sleep State Self-Rating Scale (SRSS)
Nguyen et al. 2020 [48]	Vietnam	Cross-sectional	Outpatient departments (OPD), 18–85 years (n = 3947)	February 14–March 2, 2020	Community	Prevention (behaviour change, education)	Modifications of health literacy (HL)	• Health behaviours, HL, depression, and HRQoL
Noone et al. 2020a, 2020b [52]	Global (Ireland-based)	Rapid review	Older adults	April 7, 2020	Community	Prevention	Video call interventions to usual care in nursing homes	• UCLA Loneliness Scale • Geriatric Depression Scale • Taiwanese adaptation of Short Form 36-questions health survey (SF-36)
Olagoke et al. 2020 [49]	Chicago, USA	Cross-sectional (online survey)	Public–aged ≥18years (n = 501)	March 25, 2020	Community	Prevention (behaviour change)	Social media exposure (COVID-19 news)	• Depression (Patient Health Questionnaire (PHQ-2))
Probst et al. 2020 [62]	Austria	Cross-sectional (online survey)	Psychotherapists (n = 1547)	March 24–April 1, 2020 (prior to start of pandemic)	Community	Treatment (changes in method of delivery)	Psychotherapy (changes in delivery)	• Number of appointments in person, by telephone and by internet (prior to and after Covid-19)
Rajkumar 2020 [47]	Global (India-based)	Review	Public	Not specified	Community/specialist care	Prevention and Treatment	Mental health and COVID -includes commentaries -one theme/para (based on commentaries is included on therapeutic interventions and strategies)	• Suggestions: Training community health personnel, surveys to assess scope of mental health burden in populations, online materials for education, online counselling and self-help services, telepsychiatry, services accessible to lower income
Talidong and Toquero 2020 [50]	Philippines	Cross-sectional (online survey)	School teachers (n = 218)	April 13–16, 2020	Community	Prevention (behaviour change)	Information seeking, preventive measures, and other coping mechanisms	• Psychological stress or anxiety • Practices to cope with anxiety during quarantine
Umucu and Lee 2020 [56]	Texas, USA	Cross-sectional (online survey)	Individuals with self-reported disabilities and chronic conditions (n = 269)	April 2020	Community	Prevention (behaviour change)	Coping strategies (self-distraction, denial, substance use, behavioural disengagement, venting, planning, religion, and self-blame; emotional support, humour, and religion)	• Perceived stress
Wei et al. 2020 [53]	Hangzhou, China	Prospective, randomised, controlled, 2-week study	COVID-19 patients on isolation ward (n = 26) (1) aged 18–65 years; (2) PHQ-9 or GAD-7 of ≥5; (3) completed at least a junior middle school level of education.	February 2–28, 2020	Community	Prevention (behaviour change)	Internet-based integrated intervention (incorporated breath relaxation training, mindfulness, ‘refugee’ skills, butterfly hug method)	• Hamilton Depression Scale (17-HAMD) • Hamilton Anxiety Scale (HAMA)
Zhang et al. 2020 [55]	China	Longitudinal	College students (n = 66)	February 19–March 25, 2020	Community	Prevention (behaviour change)	Physical activity	• Sleep quality • Negative emotions • Stress • Anxiety

### Collating, Summarsing, and Reporting the Results

The data from each selected study was collated and presented in Table 2. The findings were further analysed reported in the results section of the review, under the two main categories of interventions: prevention methods and treatment methods. The significance of the data was examined in the discussion.

### Consultation Exercise

Experts within the field of mental health and psychiatry were consulted early on and throughout the process to guide the research. Following Arksey and O’Malley’s recommendations, this consultation exercise was conducted in order to ‘inform and validate findings from the main scoping review’ (Arksey and O’Malley 2005). This process was instrumental in guiding the research question, provided additional references, and contributed valuable insights to guide interpretation of results including issues of effectiveness and cost-effectiveness (Arksey and O’Malley 2005).

## Results

### Search Results

The initial searches of the databases identified 804 citations after duplicates were removed. Exclusions were made to the data set in three stages (Fig. 1). After the inclusion criteria were applied to the articles, a total of 20 articles from the electronic search of databases were included in the final analysis (Fig. 1). Most studies were based on retrospective participant-reported or qualitative data, including cross-sectional (n = 8; six of which were conducted online, one via telephone, and one in person), reviews (n = 4), longitudinal (n = 2), case reports (n = 2), and qualitative (n = 2) study designs. Two studies used randomised control designs (Liu et al. 2020a; Wei et al. 2020). While randomised controlled trials have recently been initiated within the field of mental health interventions during the COVID-19 pandemic (Agyapong et al. 2020; S. Liu et al. 2020b; Moore et al. 2020; Pizzoli et al. 2020; Renjun et al. 2020), the studies have not been completed at the time of writing and so few results have yet to be published.

The majority of studies were conducted in Asia (n = 10; six from China, and one from Philippines, Vietnam, Korea, and Iraq), Europe (n = 3), and North America (n = 3). Four review articles with data from around the world were included. The sample sizes of the studies ranged from one (case report) to 4872 participants. Most studies did not specifically consider vulnerable groups; however, some analysed mental health in patients with COVID-19 or chronic diseases (n = 7), older adults (n = 4), or youth (n = 1). Age of participants ranged across the studies. Most studies examined adult populations.

### Study Populations and Settings

The specific study populations varied between studies, with most including members of the public (n = 6), older adults (n = 4), individuals with pre-existing health conditions (n = 2), individuals who had tested positive for COVID-19 (n = 5), and other specific groups (n = 3; including college students, psychotherapists, and school teachers). No studies to date have focused on interventions explicitly for individuals with prior mental health conditions.

The majority of interventions took place in a community or primary health care settings (n = 16), while others took place in hospitals (n = 3) and specialist care settings (n = 2) or focused on policy interventions (n = 1). Moreover, given that mental health concerns at different stages of the pandemic may differ, interventions were also stratified by timing of implementation. Most interventions were assessed around the peak of the pandemic in each respective country (n = 7) or the start of the pandemic (n = 5). Five articles did not specify the timing of interventions, and three others were implemented throughout the duration of the pandemic (neither at the start nor at the peak).

### Mental Health Outcome Measures

Given the broad inclusion criteria for mental health outcomes in this study, there were many mental health symptoms and disorders included in the study. The majority of studies considered anxiety or related symptoms (including panic) as the main mental health outcome (n = 9). Other studies examined depression (n = 7), overall mental well-being (n = 6), stress (n = 3), and sleep quality (n = 3).

### Current Interventions

The scoping review included any intervention that informed suggestions or treatment of mental health concerns for any population except health workers during the COVID-19 pandemic. Interventions were broadly classified into (a) those focused on prevention of poor mental health, (b) therapeutic interventions (treatment), and (c) other interventions including legislation and health system interventions (Smith et al. 2015). Most papers outlined key factors or strategies for prevention of poor mental health (n = 16), while few others discussed potential approaches to treatment in individuals with poor mental health (n = 7).

#### Prevention Methods

Some of the prevention strategies that have been explored included public health education (n = 6), reducing or changing social media exposure patterns (n = 4), technology-based interventions (n = 4), physical activity (n = 2), modifiable coping strategies (n = 2), policy adaptations (n = 2), and therapeutic preventative interventions (n = 1).

##### Public Health Education

Several of the included studies pointed to the significant amount of information about COVID-19 in the news and media, which may be a source of anxiety for the public (Ahmad and Murad 2020; Gao et al. 2020; Goodman-Casanova et al. 2020). This stems from both the quantity of information and the presence of fake news.

Providing reliable COVID-19 information sources, from government ministries and information sharing through cross-section collaborations, may assist in alleviating the anxiety and fear associated with this (Ahmad and Murad 2020; Gao et al. 2020; Goodman-Casanova et al. 2020; Nguyen et al. 2020; Rajkumar 2020). One study in particular found that increased health literacy was associated with decreased depression and increased health-related quality of life (Nguyen et al. 2020), which may be an area for future interventions to focus.

##### Social Media

Social media was also identified as a factor associated with spreading fear, anxiety, and depressive symptoms, largely due to the significant amount of information related to COVID-19 and the confusion surrounding fake news (Ahmad and Murad 2020; Gao et al. 2020; Olagoke et al. 2020). As such, many studies suggested reducing social media use, particularly in relation to accessing information about COVID-19. However, a study of school teachers in Philippines found that spending more time on social media was used as a positive coping mechanism to help teachers remain connected during physical distancing policy measures (Talidong and Toquero 2020).

##### Technology-Based Interventions

Technology-based interventions have been designed and implemented for both mental health prevention and promotion/treatment strategies. Three technology-based interventions were explored to assist older adults and prevent poor mental health, including smart phone-based applications, a TV-based platform, and video call interventions (Banskota et al. 2020; Goodman-Casanova et al. 2020; Noone et al. 2020a, 2020b). The study of smart phone-based applications identified applications to assist older adults with social interaction, facilitating telemedicine, prescription management, health and fitness apps, and apps to support visually and hearing impaired individuals (Banskota et al. 2020). Importantly, while this review analysed various applications and reported on those with highest ratings and feedback, it did not investigate the use of these applications in relation with mental health outcomes in older adults. A television platform was designed to assist older adults obtain information related to COVID-19, as a recreational activity, and perform memory exercises as an intellectual activity (Goodman-Casanova et al. 2020). Second, an Internet-based integrated intervention designed for COVID-19 patients and focusing on relaxation, self-care, and developing a sense of security was found to be significantly associated with decreased levels of anxiety and depressive symptoms (Wei et al. 2020). Finally, a review of video calls for older adults in nursing homes during prior pandemics assessed their effectiveness for preventing loneliness and depression and improving overall quality of life (Noone et al. 2020a, 2020b). The study found that video calls may result in little to no difference in the mental health outcome measures.

##### Physical Activity

Two studies identified physical activity as a potential intervention to support individuals’ mental health during the COVID-19 pandemic, one of which was targeting older adults (Goethals et al. 2020) and the other targeting college students (Zhang, Zhang, Ma, & Di, 2020).

A qualitative study of a physical activity group for older adults in France found that while online physical activity support groups are available for older adults (such as video clips), many older adults were not aware of this and were not interested in using online tools, despite recognising the need for physical activity after their programmes had been temporarily put on hold (Goethals et al. 2020). The authors highlighted that some local structures had sent physical activity advice and exercises in the form of booklets to older adults, which may have been better received.

The study of college students found that physical activity, optimally at 2500 metabolic equivalents (METs) every week, was associated with improved mental health and well-being (Zhang et al. 2020).

##### Modifiable Coping Strategies

Two cross-sectional studies analysed modifiable coping mechanisms for decreasing anxiety and stress during COVID-19. A study of schoolteachers found that respondents reported that spending time with family, seeking spiritual guidance, and talking with friends or partners online were the best ways to cope with COVID-19-related anxiety (Talidong and Toquero 2020). Another study of individuals with self-reported disabilities or chronic conditions found that modifiable coping strategies accounted for 54% of variance in well-being (Umucu and Lee 2020). Active coping, use of emotional support, humour, and religion were associated with higher well-being scores. Surprisingly, denial and self-blame were also linked to higher well-being scores (Umucu and Lee 2020).

##### Policy Adaptations

A review article and data from past pandemics found that quarantine periods have been associated with negative psychological consequences (Brooks et al. 2020). Based on past pandemics, the study suggests policy adaptions to minimise the psychological consequences for the public, including keeping quarantine as short as possible, providing the public with quality information, providing adequate supplies, and improving communication and boredom coping strategies (Brooks et al. 2020).

A study of Google search terms found that in the period immediately after COVID-19 was identified as a public health emergency, search terms related to suicidal ideation, anxiety, negative thoughts, and sleep disturbances drastically increased (‘interruption’ phase) (Jacobson et al. 2020). Soon after stay-at-home orders were introduced (‘new normal’ phase), the frequency of such search terms reduced, despite them still remaining higher than before the pandemic began. As such, the authors concluded that stay-at-home orders immediately mitigated suicide risk, although this might last only temporarily.

##### Therapeutic Interventions

One study analysed the use of progressive muscle relaxation (PMR) for patients with COVID-19 in an isolation ward and reported that PMR was found to be associated with reduced anxiety and improved sleep quality (Liu et al. 2020a).

#### Treatment Methods

Four papers identified treatment interventions for individuals who presented with mental health symptoms during COVID-19. These interventions were primarily technology based, focusing either on adapting existing treatment given physical distancing policies (n = 5), or creating new technology-based platforms to support individuals with poor mental health (n = 3; one via smartphone applications, and one via television platform). One intervention was provided in person, a case study providing dialectical behavioural therapy (DBT) in a pregnant woman being treated for COVID-19 (Huang et al. 2020). This was found to be associated with decreased symptoms of depression and anxiety.

##### Adapting Existing Treatment

Medical services have traditionally been delivered in person; however, even before the COVID-19 pandemic, there were advances in telemedicine in psychiatry (Roine et al. 2001). Telemedicine encompasses the ‘use of information and communications technology to provide health care services to individuals who are some distance from the health care provider’ (Roine et al. 2001). As a result of physical distancing, many health providers had to shift their mode of service delivery, including the provision of mental health care, in general practice, psychiatry, and psychotherapy.

One study of psychotherapists in Austria found that most patients received in-person care/psychotherapy before COVID-19 lockdown, whereas most patients were treated via telephone during the COVID-19 lockdown (Probst et al. 2020). They also found that Internet-based psychotherapy was not used as much, partially because it was not widely available in Austria at the time and there were perceived problems on behalf of the psychotherapists (technology problems, increased hassle, perceptions of impersonality) (Probst et al. 2020). Interestingly, patients had more positive attitudes towards Internet-based psychotherapy than psychologists. Other studies provided psychological first aid (Cheng et al. 2020) and telemedicine combined with mind-body modalities (i.e. mindfulness meditation) (Kwon et al. 2020) through web-based platforms. Both were provided to COVID-19 patients by physicians; immediate psychological care was provided in hospital by, while telemedicine and mind-body modalities were provided in community settings to COVID-19 positive patients.

A review article of smartphone applications to support older adults identified three medical applications to facilitate telemedicine and to connect individuals with primary care providers, psychiatrists, or psychologists via video or phone (Banskota et al. 2020). While these present promising tools to explore, the effectiveness of these applications in comparison to in-person care has been studied yet.

Lastly, a review article summarising recommendations from experts during the COVID-19 pandemic found that mental health providers may have a role in providing online counselling and self-help services, as well as providing care via synchronous and asynchronous telemedicine and telepsychiatry methods (Rajkumar 2020).

##### Creating New Treatment Programmes/Platforms

In addition to adapting existing mental health service delivery to online platforms, some interventions have been created to provide support to individuals with mental health conditions during COVID-19. One example is a television-based platform that provides both preventative and treatment support to older adults during COVID-19 (Goodman-Casanova et al. 2020). No difference was found between the control and intervention groups in this study. However, future replication of the study may be warranted as the study’s small sample size may have contributed to the occurrence of a type II error.

Another review of smartphone applications sought out smartphone applications to connect individuals with mental healthcare service providers (Banskota et al. 2020). Some of the applications provided adaptations to existing service provision (such as telemedicine), but they also provide new innovative ways of connecting older adults with service providers 24/7 through different platforms. The effectiveness of these applications has not been studied yet.

Lastly, a review of expert opinions suggested that new interventions might consider training community health personnel to provide basic mental health care to populations (Rajkumar 2020). There has not yet been any evidence regarding the implementation or effectiveness of such initiatives; however, it represents an area for possible exploration.

## Discussion

The aim of this scoping review was to examine the current evidence-based recommendations for positive mental health outcomes and well-being among the general public affected by the COVID-19 pandemic. Given COVID-19’s recent emergence worldwide, it is anticipated that many current interventions to address mental health are on-going. The use of a scoping review facilitates on-going learning through its iterative methodology that allows for continued updating given newly arising literature. This research therefore aims to provide an overview of what has currently been done and to also allow for readers to update the search based on the methodology outlined. In this way, we can provide timely and adaptive learning to the COVID-19 response, reacting to the rapidly evolving evidence-base on COVID-19, thus ensuring we are drawing on current best practice. Our findings suggest that there are many possible recommendations that may be positively associated with improved general public mental health outcomes.

### Evidence-Based Recommendations

A major challenge of this study is the lack of available evidence concerning the effectiveness of various interventions. This study aims to provide preliminary suggestions based on currently available evidence. However, the included studies were not yet able to provide a causal relationship between these recommendations and improved mental health. Despite this, the research findings may have implications for clinical practice and public health policy, until further evidence emerges.

#### Clinical Practice

One of the ways that clinicians may be able to support patients’ mental health during this pandemic is by providing health education and supporting health literacy. This may include providing accurate and relevant information to patients as well as directing them to reliable sources to remain updated on news relating to COVID-19. The literature suggests that the information overload contributed to worse mental health for many (Ahmad and Murad 2020; Gao et al. 2020; Goodman-Casanova et al. 2020; Nguyen et al. 2020; Rajkumar 2020), especially as many have obtained information about the pandemic through social media (Ahmad and Murad 2020; Gao et al. 2020; Olagoke et al. 2020). Clinicians might consider recommending that patients limit social media use to connecting with friends and family, rather than as sources of health information (Ahmad and Murad 2020; Gao et al. 2020; Olagoke et al. 2020).

The study findings also suggest that physical activity may be positively associated with improved mental health. Clinicians may consider encouraging patients to engage in daily physical activity (Goethals et al. 2020; Zhang et al. 2020). For older adults who might continue to avoid public spaces, providing exercise information may be useful (Goethals et al. 2020).

Lastly, clinicians may explore the role that technology may play in supporting the provision of cost-effective care as well as its role in prevention of mental health concerns. While past research has indicated that telemedicine is not necessarily as effective in each field, it has demonstrated effectiveness in psychiatry (Roine et al. 2001). Patients may have more positive attitudes to Internet-based psychotherapy (and possibly other mental health care options) than physicians (Probst et al. 2020). Beyond the regular provision of care delivered through technological platforms, physicians may incorporate additional treatment methods, including mind-body modalities (i.e. mindfulness meditation) (Kwon et al. 2020), and psychological first aid (Cheng et al. 2020). There are various mechanisms to provide care, including synchronous and asynchronous methods (Rajkumar 2020), which may be explored by community health workers, general practitioners, and psychiatrists. Beyond technology as a tool for treatment and providing care, its platforms may also be recommended to patients to prevent mental health issues (Banskota et al. 2020). Also, the relatively low expense associated with providing technology-based remote interventions may result in significant economic benefits, particularly regarding costs relating to the treatment of largely unprioritised mild to moderate mental health issues (Organisation for Economic Co-operation and Development (OECD) 2014).

#### Policy

Public health and policy implications of the research suggest that there are two important ways policymakers may support population mental health during the COVID-19 pandemic. This includes providing reliable and accessible sources of information relating to COVID-19 in each locality (Ahmad and Murad 2020; Brooks et al. 2020; Gao et al. 2020; Goodman-Casanova et al. 2020; Nguyen et al. 2020; Rajkumar 2020). This would be useful to combat the information overload and the difficulties many members of the public face in deciding which sources to obtain COVID-19-related information from. Second, the mental health impacts of quarantine may be reduced by ensuring that (a) quarantine measures are enacted quickly (Brooks et al. 2020) but kept as short as possible, and (b) individuals are provided with adequate information, supplies, and communications during this time (Brooks et al. 2020; Jacobson et al. 2020).

## Limitations

While great effort was extended to extract as much information as possible from the currently available evidence, we find that this topic remains under-reported in literature. Given the fast-paced nature of the COVID-19 pandemic, there remains a lag between intervention implementation, research, and publication, which results in a lack of current literature to definitely analyse which interventions have been most effective at preventing and treating mental health issues during the pandemic. This was addressed by handsearching COVID-19 databases for additional peer-reviewed studies. Additionally, near the end of the review process, the databases were briefly re-searched for any additional studies that may have come out throughout the review process. Despite this, there remains a lack of evidence regarding the effectiveness of interventions to improve mental health during the COVID-19 pandemic. Because there have not been any rigorous RCTs published on this topic, the review was limited to qualitative, cross-sectional, and longitudinal studies, meaning that it is not possible to infer causal relationships between exposure to interventions and mental health outcomes.

Moreover, there was a great deal of heterogeneity between studies, given variances in types of interventions, mental health outcome measures, intervention settings (i.e. in community settings, clinical settings, policy making implications), and timing of interventions (i.e. at the start, middle or plateau stages of the pandemic). All of these are factors that could influence the outcome variables, and therefore make comparisons of the various interventions that have been implemented thus far difficult.

Finally, most included studies did not report on differences based on factors such as gender, race, age, socioeconomic status, and prior mental health considerations, which are important for equity considerations. While the purpose of this study was to consider the mental health impacts and interventions that have been impacted thus far from a general public health perspective, future research in this field should ensure that equity considerations are incorporated into mental health interventions and research. This may be done in the future by focusing on interventions implemented within specific sub-populations.

## Conclusions

Given the increase in mental health concerns during COVID-19 (Gao et al. 2020; Steven Taylor 2019), which is not expected to resolve quickly on its own (Steven Taylor 2019), it is essential that more research be conducted in order to understand what interventions may be implemented to support population mental health.

As there are numerous notable randomised controlled trials in this area that are expected to come out (Agyapong et al. 2020; Cheng et al. 2020; Chen 2020a, 2020b; Liu et al. 2020c; Moore et al. 2020; Pizzoli et al. 2020; Renjun et al. 2020), a future review could focus specifically on novel effective interventions to address mental health issues during the pandemic.

Moreover, more work can continue to identify vulnerable populations. These may include both those at risk for new mental health issues given the circumstances surrounding COVID-19, and also individuals who had already been suffering from mental health issues requiring psychiatric care prior to COVID-19. Research designed to assess the effectiveness of both prevention and treatment interventions, especially considering changes in treatment given COVID-19 would be valuable in guiding clinicians and policy makers.

## Declarations

### Human/Animal Studies

No animal or human studies were carried out by the authors for this article.
